# Do Executives' Backgrounds Matter to IPO Investors? Evidence from the Life Science Industry

**DOI:** 10.1371/journal.pone.0060911

**Published:** 2013-05-15

**Authors:** Jay Chok, Jifeng Qian

**Affiliations:** Keck Graduate Institute, Claremont, California, United States of America; Old Dominion University, United States of America

## Abstract

In this study, we focus on the impact of senior executives' industry backgrounds on the amount of capital raised in the stock market. The primary contribution of the study entails applying the upper echelon theory to the initial public offering (IPO) phenomenon. Specifically, we hypothesize that the industry backgrounds of corporate executives affect the amount of capital that the firm raised in the primary stock market. We argue that the firm's future investment strategies are unobserved by the investors ex-ante and investors expect firms' investment strategies to be based on the executives' industry backgrounds. As a result, the executives' industry backgrounds influence the investors' expectations about what investment strategies the firm is likely to deploy. Furthermore, the above logic also suggests that executives of different industry backgrounds should prefer different investment strategies corresponding with demand for different amount of capital. As a result, we expect the industry backgrounds to covary with the capital raised from both the supply and demand perspectives. To test the hypotheses, we ran a reduced econometric model wherein the executives' background predicts the amount of capital raised. Regression analyses suggest that the capital raised is negatively associated with the number of senior executives with prior career experience in the healthcare and genomic sectors but positively associated with the number of senior executives with prior career experience in regulatory affairs. The results provide tentative support for the notion that investors infer corporate strategies from senior executives' industry backgrounds.

## Introduction

What do investors buy when they invest in start-up firms with no substantial assets-in-place? The short answer to the first question is that the investors are buying growth options. And why would the firm's demand for capital vary with its growth options? The short answer to the second question is that the firm raises capital to finance growth.

But investors do not know ex-ante which investment strategies that the firm will deploy and what growth options will be exercised as a result of those investment strategies. Upper echelons theory states that corporate strategies are influenced by the psychological make-up of the top management team [1]. Prior researchers have tested variables, such as demographic characteristics, education, age, financial position, as proxies for the psychological make-up of the top management team (Table 1). In this paper, we conjecture that investors infer the likely investment strategies and growth options that the firm will exercise based on the industry backgrounds of the firm's executives. Likewise, if the corporate executives' industry background affects the type of investment strategies and growth options a firm is likely to exercise, we should expect industry backgrounds to correlate with capital raised. We test this conjecture by applying the upper echelon theory to the initial public offering (IPO) phenomenon. Specifically, we hypothesize that the proceeds from an IPO are affected by the industry backgrounds of corporate executives. In the subsequent paragraphs, we first explain the upper echelon theory and discuss the relationships that have been tested in this theory. This is followed by a discussion of prior research on IPOs. In prior research, financial economists had focused mainly on asset mis-pricing and did not apply the upper echelon theory to understand how corporate executives' industry backgrounds might affect IPO proceeds. We then define what growth options are and discuss why using corporate executives' industry backgrounds to make inferences about the firm's growth options (along with the investment strategies) should affect the amount of capital raised. Finally, we develop specific hypotheses related to corporate executives' industry backgrounds.

**Table 1:** Summary of the tested relationship between the top managers' characteristics and organizational performance.

Literature	Top managers' characteristics	Organizational performance
Hambrick &Mason [85]	Demographic characteristics	Strategies
Haleblian & Finkelstein [86]	Top management team size and CEO Dominance	Profits in turbulent Environments
Jackson et al. [87]	Group Heterogeneity	Group performance
Hambrick and Mason [85]	Socio-economic background	Growth and profit variability
Carlson [88]	Career experiences	Changes in structure Procedure, and people

Prior research on IPOs has focused almost exclusively on underpricing and long run underperformance as evidenced by the comprehensive survey [2]. Many underpricing studies simply assume that by minimizing underpricing, the IPO proceeds would be maximized [3], [4], [5]. However, high prices prevailing immediately after the issue date may not necessarily be the result of underpricing but could be due to overvaluation, fads in the aftermarket [6] and the existence of underwriter support [7], [8]. Moreover, the measure of initial return could have been overly simplistic as it overestimates the initial returns available to the investors and the costs of underpricing to the issuer [9]. In fact, Purnanandam and Swaminathan [10] document that overvalued IPOs earn especially high first-day returns and low returns over the next five years. This is consistent with long run underperformance research, which indicates that underpricing is sensitive to the length of time period being examined [11], [12].

Further, underpricing and proceeds might be positively correlated in certain instances. Recent research (i.e. book building theories) posits that IPO issues that suffer from ex-ante mis-pricing can increase proceeds via the partial adjustment phenomenon [13]. The partial adjustment phenomenon exists because investment bankers will reward institutional investors for revealing information about the true value of the firm during the IPO road shows [12]. Because any increase in offer price during the book building period is a fraction of the full adjustment that should have taken place, using price discovery during the IPO road shows to maximize IPO proceeds might result in greater underpricing [13], [14].

Indeed, not only is it impossible to know ex-ante whether an issue would be underpriced or overpriced but we also cannot assume that minimizing “underpricing” can maximize the IPO proceeds [9]. Thus, an independent study of IPO proceeds is warranted. This theoretical gap is first filled by management scholars. Specifically, Deeds, Decarolis and Coombs [15] tested whether the capabilities of biotechnology firms act as signals to the institutional investors. They argued that biotechnology firms signal strong firm capabilities to overcome information asymmetry and adverse selection. The indicators of strong firm capabilities include being located in biotechnology clusters, having more patents and scientific papers that are highly cited, and having more products. They found that these indicators are positively associated with IPO net proceeds Nonetheless, signaling cannot fully explain firms' behavior [15]. Therefore, the question as to what is the intrinsic reason why institutional investors invest in a firm is left unanswered.

We conjecture that the proceeds from an IPO are positively related to the growth options embedded in young companies. We define a growth option as the right but not the obligation to invest capital in a productive asset [16]. According to Myers [17], firm value consists of the assets-in-place and growth options. However, Myers [18] subsequently coined the term “real options” and as such, growth options came to be used either interchangeably with real options or considered as a subset of real options. In this paper, we use growth options and real options interchangeably.

Our conjecture with regard to the relationship between IPO proceeds and growth options is supported by prior research, which suggests that firm value and growth options are positively correlated. That is, a firm goes public to finance growth, which can increase firm value. Further, investors may invest in an IPO in order to purchase growth and the rationale for purchasing growth is to buy into companies that will experience increase in firm value [19]. From the supply perspective, the IPO volume (the number of firms going public in a given year) appears to be dependent on the firms' demand for capital [20]. Moreover, recent surveys of CEOs and CFOs indicate that the most important reason for going public is to infuse a significant amount of investment capital into the firm to finance growth [19]. From the demand perspective, it can be argued that investors' demand for growth options might be intimately related to firm value. Specifically, growth options and firm value might be positively correlated under certain conditions. In theoretical research, Pindyck [21] shows mathematically that growth options represent more than half of a firm's value if demand volatility exceeds 0.2. Berk, Green and Naik [22] developed a model and ran simulations to show that growth options affect security returns, which affect firm value. Willner [23] presents a jump formulation to value a start-up enterprise as a real compound option.

In empirical research, Kester [16] estimates that, on average, the value of a firm's growth options is more than half of its market value. In particular, the value of growth options is about 70 to 80% of a firm's market value in industries with high demand volatility. Subsequent empirical studies also provide statistical results that suggest a significant portion of the market value of equity is accounted for by growth opportunities [24]. Overall, it seems that the idea that investors value growth options and reflect it in the market value of a firm appears to be well supported by both theoretical and empirical research.

To test this theory, our sample consists of the broader life science industry since the industry heterogeneity ensures that firms pursuing different investment strategies would have different levels of growth options. For example, biotechnology companies are perceived to be associated with high levels of uncertainty [25], [26]. The implication is that growth options should be more valuable in these companies. Indeed, both academic and practitioners' insights show that biotechnology companies are likely to consist of mainly growth options rather than assets-in-place [26]. As such, it is perceived to be appropriate to value biotechnology companies as growth options [27], [28].

On the other hand, the revenue of a healthcare company tends to be more predictable because (1) majority of their clients are covered by reimbursement plans and (2) reimbursement limits are imposed [29]. Moreover, individuals will rationally adjust their demand for healthcare to reduce uncertainty associated with medical expenditures [30].

Finally, genomic tools companies are regarded as the life science equivalent of the dot-com plays because history shows that genomic companies simply cannot grow their revenue exponentially [31]. The nature of the business, wherein potential revenue is to come from selling genomic tools and subscription services for genetic information, is less volatile compared to drug development but is unlikely to result in more growth options [32]. Further, it can be argued that the growth prospects of genomic tools companies had generally been hyped up. This has allowed genomic tools companies to use the “pick and shovels” theory to present themselves as firms with many growth options [32]. Preemptive patenting by genomics firms, which camouflaged the fact that these are speculative ventures with no growth options, had caused some initial alarm although genomic firms do not necessarily engage in business opportunities with growth options such as the development of drugs [33]. Thus far, four speculative events have unraveled, which provided us with the hindsight to see genomic companies with a proper perspective [34]. However, since institutional investors are allocated the bulk of an IPO and given their information advantage, it should be expected that sophisticated investors will see through the hype [13], [14]. Extant empirical research supports such a stance [35], [36].

It is also possible that investors may invest in an IPO in order to profit from the growth options associated with extracting resources from the government (for further reference, see Chok [37], [38], [39], and Pfeffer and Salancik [40]). For example, pharmaceutical companies may be able to influence the amendments to patent laws by lobbying the government [41]. Amendments to the patent laws may allow the pharmaceutical firms to enhance their growth options, which have the effect of increasing profitability. In fact, our analysis of the risk factors in the IPO prospectuses in the broader life science industry suggests that the legal and regulatory affairs executives may be able to affect the returns on investments by exploiting regulations related to the FDA and the Patent Office (in areas such as clinical trials management and intellectual property licensing) and defending attacks related to patent infringements and liability (product and professional) claims. Thus, it makes sense to relate the ability to extract resources from the government to growth options.

In summary, we rely on upper echelon theory to identify proxy variables. Specifically, the industry backgrounds of senior manager and directors are expected to influence the investment strategies of the firm [42] while different investment strategies create varying amount of real options [43]. Finally, growth options and IPO proceeds should be positively correlated given that investors are investing mainly in growth options. Thus, the industry background of executives, investment strategies, real options, and IPO proceeds actually form a chain. The four industry backgrounds we identified are healthcare, genomic/analytical, biotechnology, and regulatory. Empirically, we found statistical support for three of the investment strategies (healthcare, genomic, and regulatory).

We view the life science industry as composed of four sectors: namely pharmaceutical and biotechnology, analytical and genomic tools, medical devices and supplies, and healthcare. An analysis of the industry publications and the risk factors show that managed care dominates the life science industry. In fact, the domination of HMOs has prompted calls for reforms even from business academics [44]. Hence, our industry analysis put the healthcare sector at the center stage.

**Figure 1:**
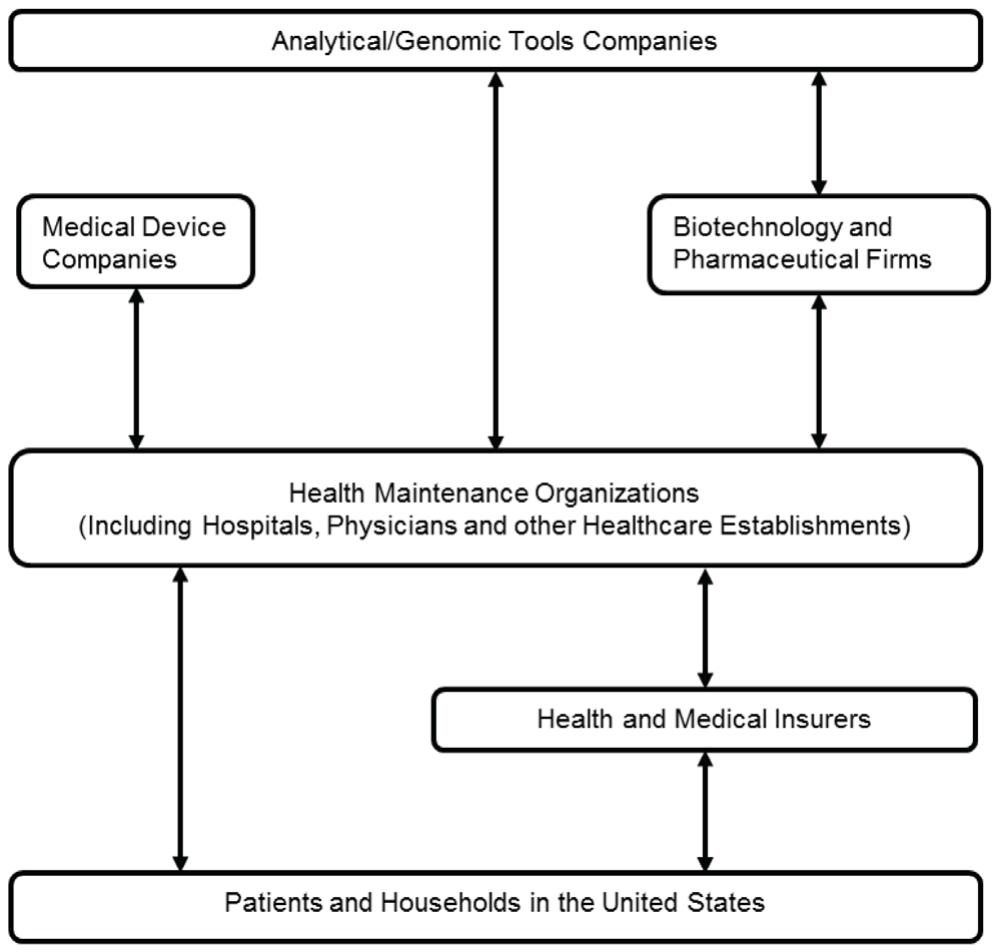
The flow of transactions in the life science industry. The arrows indicate the exchange of transactions between the sub sectors of the life science industry. In effect, that means the flow of payment and the flow of services and goods. The dominant flow of payments from the patients to the hospitals, physicians and other healthcare establishments is mediated by insurance companies and health maintenance organizations (HMO). At the same time, the flow of services from life science companies in the upstream to the patients is mediated by the hospitals, physicians and other healthcare establishments.

We show that health insurers and Health Maintenance Organizations (HMO) (see footnote 1) are in a powerful position due to the nature of their business (Fig. 1). Recent mergers and acquisitions, which reduce the number of players in the marketplace, give insurers and HMOs unprecedented clout in pricing and coverage [45]. However, consolidation is almost a sign of the dearth of growth options. In fact, even while health insurance premiums are experiencing increases of 7% to 12%, insurers and HMOs are trying to slow down the growth in healthcare spending [45].

In contrast, demand for drug delivery systems in the US, consisting of specialized dosage formulations as well as medical supplies, devices and equipment, is forecast to increase in value at over 8.5% annually (outstripping GDP growth) reaching a value of $48 billion by 2007. Finally, the prospects of the genomic sector were artificially inflated due to the influx of new biotech firms [31]. When the biotechnology boom ended in 2000, the demand for genomics products starts to fall [31].

Having described the industry, we now turn to the identification of proxy variables using upper echelon theory. Hambrick and Mason's central thesis [46] is that demographic variables can predict strategic choice because the organization is a reflection of its top managers. That is, managerial perceptions influence strategic decision making. In turn, managerial perceptions, which are shaped by the manager' cognitive base and values, are closely associated with one's demographic characteristics (see footnote 2). Our proxy variables (industry backgrounds) are related to the study of managerial functional backgrounds. Empirical evidence show that managerial functional backgrounds are associated with prospector and defender business strategies [47], [48]. Experimental evidence also shows that executives' interpretation of a complex business problem is positively associated with their departmental affiliations with statistical significance [49]. It is reasonable to assume that this is applicable to industry background. Thus, we argue that the investment strategy of a firm will be influenced by the industry backgrounds of the executives. We define an investment strategy as the choice to invest and move into a particular product market. Thus, we argue that firms with more directors and senior management from (1) the healthcare industry are more likely to exercise growth options that result in business opportunities related to healthcare, (2) the genomic/analytical industry are more likely to exercise growth options that result in business opportunities related to bioinformatics and, (3) the pharmaceutical industry including the physicians are more likely to exercise growth options that result in business opportunities related to biotechnology.

We further argue that when an investment strategy is associated with a higher level of volatility, its' real option value is higher. This is because when a firm enters into a product market with a higher level of demand volatility, the increased volatility in the product market can be translated into a higher stock return's volatility [50]. Further, real options constitute an increasing portion of firm value as the stock return's volatility increases [24], [51]. Recent research validates this idea empirically by documenting a significant negative relationship between investment to capital ratio (a proxy for product market volatility) and idiosyncratic stock return volatility [52].

In addition, the real options literature generally assumes that investors will account for real options in their investments [53]. Empirical work in real options also adopts such a stance. Quigg [54] documents that option-based land valuations are better approximations of market land prices and, Berger, Ofek and Swary [55] provide empirical evidence that the stock market values the abandonment option. Thus, if IPO investors are indeed sophisticated and informative [13], [14], we expect IPO proceeds to correlate with the executives' industry backgrounds. The notion that industry backgrounds matter leads us to develop a set of hypotheses as discussed below.

The overarching null hypothesis is that industry backgrounds do not matter for IPO proceeds. The alternative hypothesis is that industry backgrounds do matter for IPO proceeds. We first consider hypotheses related to healthcare and biotech industry backgrounds. Prior research generally assumes that the investment moves of biotech firms lead to business opportunities with greater volatility and growth whereas hospitals do appear to have fewer growth options as a result of limited revenue growth [27], [45]. In fact, when we compare the volatility of three healthcare exchanged traded funds (see footnote 3) with a biotech exchanged (see footnote 4) traded fund, we find that healthcare exchanged traded funds are less risky than the biotech exchanged traded funds (see footnote 5). If biotechnology stocks are more volatile compared to healthcare stocks, we could infer that a biotechnology investment strategy is associated with a higher value of real options relative to a healthcare investment strategy. Moreover, the growth prospects for drug companies also appear to be higher than the healthcare companies based on the difference between the Fama-French three-factor model (cost of equity) and the CAPM (cost of equity). Specifically, Fama and French [56] interpret the difference between the Fama-French three-factor model (cost of equity) and the CAPM (cost of equity) as the degree to which the stock market rewards the firms for their growth prospects. In particular, the difference between these two measures of the cost of equity for drug companies is 4.62 whereas the difference for health services providers is 1.81 were reported [56]. Therefore, we hypothesized that

H1 (Null): IPO proceeds are not associated with the number of top managers with the healthcare industry background.

H1 (Alternative): IPO proceeds are negatively associated with the number of top managers with the healthcare industry background.

H2 (Null): IPO proceeds are not associated with the number of top managers with the biotechnology industry background.

H2 (Alternative): IPO proceeds are positively associated with the number of top managers with the biotechnology industry background.

No genomic exchange traded fund exists and it is also not easy to differentiate between pure genomics firms and those firms that intend to use genomic tools to develop drugs. Some genomic firms may evolve into therapeutic product companies whereas other genomic firms may evolve into information providers or tools companies. Nonetheless, pure genomic firms do not have the potential to realize big sudden cash flows because they do not develop drugs [32]. As such, pure genomic firms are like dot-com plays [31], [34]. Thus, we hypothesized that

H3 (Null): IPO proceeds are not associated with the number of top managers with the genomic/analytical industry background.

H3 (Alternative): IPO proceeds are negatively associated with the number of top managers with the genomic/analytical industry background.

We also argue that executives with legal and/or regulatory backgrounds are more likely to conceive regulatory strategies to exercise resource dependence on the government. It has been showed that in a nationally regulated industry, the proportion of corporate elites with a legal and regulatory background should be negatively associated with idiosyncratic volatility for biotech IPO firms [57]. This seems likely because regulatory expertise form the cognitive base and values of legal/regulatory executives and it has been shown that legal executives bargain strategically in order to extract policy concessions from other stakeholders [58], . This ability to extract resources from the legal and regulatory system can be seen as a real option to exercise resource dependencies on the government. For example, Hirsch [41] compares the phonograph record and the pharmaceutical industries, and concludes that the difference in profits could be traced to the management of resource dependencies. Specifically, pharmaceutical companies would engage in activities designed to modify patent laws, which increase the value of their real options. Dunford [61] also utilize the resource dependence theory to explain how technology could be suppressed via legal strategies. In effect, this creates a type of delay option for the firm since it has the right but not the obligation to use the technology. Therefore, we hypothesize that

H4 (Null): IPO proceeds are not associated with the number of corporate executives with the legal/regulatory industry background.

H4 (Alternative): IPO proceeds are positively associated with the number of corporate executives with the legal/regulatory industry background.

## Materials and Methods

### Statistical model

Data is collected from both SDC and the IPO prospectuses. IPO prospectus are available from Edgar since early May 1996 [62]. Thus, for ease of data collection, our sample period is defined as 1^st^ May 1996 to 31^st^ Dec 2001. We downloaded data on life science companies from SDC. These two criteria resulted in 290 companies. The sample size is reduced to 245 based on IPO prospectus availability on Edgar. We remove 5 outliers (see footnote 6). This reduced the sample size to 240 companies. The representative econometric specification for the variables with IPO net proceeds as the dependent variable is shown below.
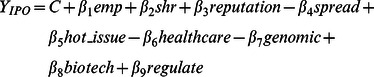
(1)Where 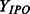 is log of IPO net proceeds; C is a constant, emp is the log of number of employees; shr is the log of number of shares locked up; reputation is the log of underwriter reputation; spread is gross spread (percentage) and is measured as the difference between IPO gross proceeds and IPO net proceeds over the IPO gross proceeds; hot_issue is a dummy for hot issue; healthcare is the number of senior managers and directors with the healthcare industry background; genomic is the number of senior managers and directors with the analytic/genomic industry background; biotech is the number of senior managers and directors with the physician or pharmaceutical industry background; regulate is the number of senior managers and directors with the regulatory industry background.

We log the dependent variable: IPO net proceeds. We also log the control variables if the distribution is skewed.

### Explanatory variables

The four explanatory variables are about the industry background of senior managers and directors. That is, we combine the senior management and the board of directors to form our measures of corporate executives. To code the necessary data, we review the career histories of close to 3000 senior managers and directors in 245 IPO prospectuses. The Securities and Exchange Commission (SEC) requires the IPO documents to provide biographical and career information of the senior executives (i.e. managers and directors) for the past five years before the IPO filing date. The IPO firms often provide more historical information than the five-year rule. We coded the executives' industry background based on the biographical information provided. The coding of industry background is not mutually exclusive. However, when an executive has experience in more than one industry category but focus primarily on one sector, we do not code the executive's miscellaneous backgrounds. Although it is possible for an executive to have more than one primary industry background, we find that many executives' recent background is generally focused on a single broad sector. This makes sense since an executive generally has limited time and therefore need to focus in a certain sector. A senior manager or director is coded as healthcare if he or she has experience in selected areas such as home health care services, hospitals, outpatient care centers, nursing and residential care facilities, and HMOs and health/medical insurers.

A senior manager or director is coded as analytical or genomic tools if he or she has experience in selected areas such as medical and diagnostic laboratories, analytical laboratory instrument manufacturing including industrial lenses, and computer related work such as software, hardware and semiconductors.

A senior manager or director is coded as biotechnology if he or she is professionally trained as a physician or have related training. This is because we associate directors and senior managers with physician backgrounds with clinical trials and drugs i.e. business opportunities with more growth options. We also coded a senior manager or director as biotechnology if he or she has experience in the pharmaceutical industry. The experience generally relates to the marketing or manufacturing of drugs. But it also includes general managerial experience in the pharmaceutical/biotechnology sector. Thus, our proxy for this investment strategy utilized (combine) both the physician and pharmaceutical industry backgrounds into a single variable.

A senior manager or director is coded as regulatory if he or she is a lawyer or an individual with experience with regulatory work. Regulatory work includes clinical trials management and other FDA related issues, intellectual property management including patents applications, infringements and licensing, and legal/regulatory compliance including management of product and/or professional liability claims.

### Control variables

The control variables are firm size, underwriter reputation, the number of shares locked up, a dummy for hot issue and the gross spread (in percentage terms).

Firm size is defined as the number of employees (see footnote 7). The number of employees is downloaded from SDC. Firm size is used to proxy for information asymmetry because in prior research, many researchers suggest that information asymmetry is likely to be more severe for small firms [63], [64]. Pagano, Panetta and Zingales [64] also discuss about how firm size might be related to information asymmetry. Specifically, firm size could be related to the costs of preparing information and/or reputation. Lerner, Shane and Tsai [25], also contend that the data supplied by larger firms is “cleaner”. Felo [65] argue similarly; the existence of any fixed financial reporting costs may result in lower costs as a fraction of firm size. That is, firm size and information asymmetry is negatively associated. This results in a positive relationship between firm size and reporting quality since larger firms can afford to incur the costs for high quality information disclosure. Indeed, Lang and Lundholm [66] find a positive relationship between firm size and AIMR scores, which proxy for disclosure quality. Korajczyk [62] also documents the impact of firm size in the timing of a seasoned equity offering (SEO). Specifically, lager firms may face less adverse selection problems. Since information asymmetry might affect the amount of outside equity raised, we control for firm size.

We use a ranking table provided by Jay Ritter [67] as a measure of underwriter reputation (see footnote 8). The lead underwriter of the issuer is coded according to the ranking table and used as a variable. Due to adverse selection, IPO issuers require the help of intermediaries such as underwriters and/or venture capitalists in order to go public successfully [68], [69]. Since reputable underwriters can attenuate adverse selection problems, they can affect the amount of outside equity raised. That is, underwriter reputation is positively associated with IPO proceeds and ought to be included as a control variable.

The “number of shares locked up” is downloaded from SDC. We use the “number of shares locked up” variable as a proxy for control of agency problems. The rationale is that outside investors would be reasonably concerned about the number of shares that insiders owned and locked up, which is a more effective, albeit temporary, control mechanism. Insider ownership just prior to IPO is transient if it is not locked. This is consistent with prior research on IPO lockups. For example, Lilienfold-Toal [70] associates lockup agreements with the free riding effect and mitigation of agency problems. A model is formalized wherein stock market participants rely on larger shareholders to monitor the agents. Further, lockup agreements provide temporary guarantee of block shareholdings. As such, the expiry of lockup agreements results in negative abnormal returns [71], [72], [73]. Therefore, it is possible that the number of shares locked up is positively associated with IPO proceeds.

We control for the hot issue market and code a transaction as 1 if it occurred in 1999 or 2000 and 0 if otherwise. The business rule in Wall Street appears to be that anyone can go public in a bullish market whereas it is near to impossible to go public in a bearish market [19]. Indeed, Ibbotson and Jaffe [74], and Ritter [75] document that a clustering of a large number of IPO offerings exists during the 1960s hot issue market and in 1980 respectively. Lowry [20] documents that, at the macroeconomic level, the aggregate amount of IPO proceeds tend to be higher during such hot issue periods. Within the life science industry, such a hot issue period or financing window exist in the latter part of 1999 and the whole of 2000 [76]. Further, Hall [77] remarks that investors are highly optimistic about the growth options of start-ups when NASDAQ and NYSE indices are climbing to record levels. Thus, it can be expected that firms might raise more cash from the stock markets during hot issue markets [25].

We divide the difference between IPO gross proceeds and IPO net proceeds by IPO gross proceeds to compute a measure of gross spreads. Gross spreads could proxy for information asymmetry problems if it is cheaper to certify a high information quality firm. This argument is supported by the certification hypothesis, which posits that underwriter compensation will be higher for firms that suffer from information asymmetry and are more likely to be affected by adverse selection problems in the market [69]. This is because for third party certification to be successful or economically valuable, the services of the certifying agent must be costly for the issuing firm to obtain and the cost structure must be such that a separating equilibrium is achieved between high and low information quality firms [69]. It is therefore reasonable to assume that it is cheaper to certify a high information quality firm. Moreover, Megginson [69] finds that higher quality underwriters charge lower gross spreads, which suggest that reputable underwriters would rather certify firms with low information asymmetry problems due to the lower certification cost. Thus, given that gross spreads might be negatively associated with IPO proceeds, we control for gross spreads in the model.

## Results and Discussion

### Descriptive statistics

In this section, we describe the sample characteristics. Our life science industry sample is composed of four sectors: pharmaceutical and biotechnology, analytical and genomic tools, medical devices and supplies, and healthcare. In our sample, 127 IPOs are in the pharmaceutical and biotechnology sector, 26 IPOs are in the analytical and genomic tools sector, 63 IPOs are in the medical devices and supplies sector, and 24 IPOs are in the healthcare sector.

In Table 2, we present the standard statistical measures (mean, median, standard deviation, 25^th^ percentile, 75^th^ percentile, minimum and maximum) for the number of employees, IPO net proceeds, firm age, the educational qualifications of the senior managers and directors or corporate executives, the number of venture capitalists on the board, the percentage of firms listed in the hot issue years (1999 or 2000), venture capital invested, the number of shares locked up and gross spread.

**Table 2:** Firm Characteristics by sector.

Sector	Variable	Mean	Median	Std. Deviation	Percentile 25	Percentile 75	Min	Max
Analytical and Genomic Tools	Number of employees	166.85	60	353.671	36.75	182	0	1850
	IPO Net Proceeds (in Mil)	38.59	31.22	28.63	17.81	56.73	6.58	136.11
	Firm age	5.32	4.78	3.68	2.31	6.99	0.20	16.77
	JD_PHD_MD	4.85	4.5	3.233	3	7	0	11
	Number of VCs	1.73	2	1.402	0	3	0	4
	Hot Issue (Percentage)	0.35	0	0.485	0	1	0	1
	Venture capital invested (in mil)	25.90	14.90	30.93	0.00	40.74	0.00	129.12
	Number of shares locked up (mil)	7.41	4.28	8.34	0.00	12.68	0.00	25.09
Healthcare	Number of employees	629.92	79	1671.776	5	8100	30.25	424.25
	IPO Net Proceeds (Mil)	41.69	29.84	33.98	4.63	132.92	14.19	61.69
	Firm age	5.50	4.81	3.42	0.55	11.57	2.38	8.05
	JD_PHD_MD	3.96	3	2.896	0	10	2	6.75
	Number of VCs	1.92	2	1.613	0	6	0.25	3
	Hot Issue (Percentage)	0.33	0	0.482	0	1	0	1
	Venture capital invested (in mil)	30.27	14.14	41.51	0.00	168.60	0.00	51.23
	Number of shares locked up (mil)	6.36	2.42	10.81	0.00	39.07	0.00	7.24

Sector	Variable	Mean	Median	Std. Deviation	Percentile 25	Percentile 75	Min	Max
Medical Device	Number of employees	328.69	102	515.493	0	2400	51.5	322
	IPO Net Proceeds (in Mil)	43.65	33.25	33.86	5.98	161.82	18.38	52.00
	Firm age	6.86	5.50	5.66	0.48	30.76	2.79	8.20
	JD_PHD_MD	4.33	3	3.448	0	12	1	7
	Number of VCs	1.59	2	1.346	0	4	0	3
	Hot Issue (Percentage)	0.31	0	0.467	0	1	0	1
	Venture capital invested (in mil)	38.39	18.72	47.28	0.00	218.65	0.00	58.97
	Number of shares locked up (mil)	7.99	4.46	10.98	0.00	51.86	0.00	10.45
Pharmaceutical and Biotechnology	Number of employees	827	120	3832	3	42000	61	301
	IPO Net Proceeds (Mil)	60.62	36.27	152.80	2.44	1723.50	22.19	60.91
	Firm age	7.44	5.35	7.58	0.55	51.15	3.78	8.09
	JD_PHD_MD	4.43	5.00	3.31	0.00	14.00	2.00	6.00
	Number of VCs	1.88	2.00	1.53	0.00	6.00	0.00	3.00
	Hot Issue (Percentage)	0.40	0.00	0.49	0.00	1.00	0.00	1.00
	Venture capital invested (in mil)	37.75	21.50	49.29	0.00	361.38	0.00	54.26
	Number of shares locked up (mil)	8.57	6.65	9.95	0.00	48.30	0.00	14.35

Firm age is computed as the difference between the founding date and IPO date. JD/Ph.D/MD refers to the number of corporate elites in the firm with a Juris Doctorate, Medical doctorate or Ph.D. Regulatory expertise refers to the number of corporate elites with legal training and/or experience in regulatory work. Number of VCs refers to the number of venture capitalists sitting on the board of directors. Venture capital invested is the total amount of dollars invested by venture capitalists in the firm. Number of shares locked up refers to those shares owned by insiders that cannot be sold within a stipulated holding period. We divide the difference between IPO gross proceeds and IPO net proceeds by IPO gross proceeds to compute a measure of gross spreads.

40 percent of the pharmaceutical/biotechnology firms and 35 percent of the analytical/genomic tools companies chose to go public during the hot issue years (1999 or 2000). 33 percent of the healthcare companies and 31 percent of the medical device companies went public during the hot issue years. Thus, relative to the other sectors, biotechnology firms' willingness to go public appears to be most influenced by financing windows [25], which probably explains why, on average, firms in the pharmaceutical and biotechnology sector raise more cash relative to the other sectors. Specifically, the average IPO net proceeds is 60.62 million dollars in the pharmaceutical and biotechnology sector, 43.65 million dollars in the medical device and supplies sector, 41.69 million dollars in the healthcare sector and, 38.59 million dollars in the analytical and genomic tools sector.

Because biotechnology firms have alternative ways of financing growth when the stock market is bearish, they are more likely to time their equity issue [25], [78]. This “timing” of equity issue probably explains why firms in Pharmaceutical & Biotech sector went public at a relatively older age compared to the other sectors. On average, firms in the Pharmaceutical & Biotech sector went public at 7.44 years old. This is followed by 6.86 years old in the Medical Device sector, 5.5 years old in the healthcare sector and 5.32 years old in the Analytical & Genomic sector.

There appears to be no discernible trends between the number of venture capitalists sitting on the board and the venture capital invested. On average, there are 1.59 venture capitalists sitting on the board in the Medical Device sector and the venture capital invested is 38.39 million dollars. There are 1.88 venture capitalists sitting on the board in the Pharmaceutical & Biotech sector and the venture capital invested is 37.75 million dollars. There are 1.73 venture capitalists sitting on the board in the Analytical & Genomic sector and the venture capital invested is 25.90 million dollars. There are 1.92 venture capitalists sitting on the board in the healthcare sector and the venture capital invested is 30.27 million dollars.

On average, the number of shares locked up is 8.57 million shares in the Pharmaceutical & Biotech sector, 7.99 million shares in the Medical Device sector, 7.41 million shares in the Analytical & Genomic sector and 6.36 million shares in the healthcare sector. The average number of employees is 827 in the Pharmaceutical & Biotech sector, 167 in the Analytical & Genomic sector, 329 in the Medical Device sector and 630 in the healthcare sector. The average number of corporate executives with Juris Doctorate, Medical Doctorate or Ph.D is 4.43 in the Pharmaceutical & Biotech sector, 4.85 in the Analytical & Genomic sector, 4.33 in the Medical Device sector and 3.96 in the healthcare sector.

### Regression analysis

We first examine the correlation matrix and then conduct the regression analysis. We fail to reject the null hypothesis that the number of senior executives with the biotechnology industry background and IPO proceeds are uncorrelated. All other null hypotheses are rejected. To ensure robustness, the regression model is subject to sensitivity analysis and the adjusted r-square remains qualitatively unchanged.

Table 3 presents the standard statistical measures (mean, median, standard deviation, 25^th^ percentile, 75^th^ percentile, minimum and maximum) for the dependent variable and the independent variables.

**Table 3:** Descriptive statistics.

Variables	Mean	Median	Std. Deviation	25 Percentiles	75 Percentiles	Min	Max
**Dependent Variable**							
Log of IPO Net Proceeds	1.51	1.52	0.34	1.27	1.73	0.39	2.32
**Explanatory Variables**							
Healthcare	0.16	0.00	0.83	0.00	0.00	0.00	7.00
Genomic	0.21	0.00	0.79	0.00	0.00	0.00	6.00
Biotechnology	1.37	1.00	1.37	0.00	2.00	0.00	6.00
Regulatory	0.90	1.00	0.99	0.00	1.00	0.00	4.00
**Control Variables**							
Log of Number of Employees	2.11	2.00	0.63	1.73	2.42	0.00	3.97
Log of Number of Shares Locked Up	4.52	6.67	3.32	0.00	7.09	0.00	7.71
Log of Underwriter Reputation	0.86	0.91	0.17	0.85	0.96	0.04	0.96
Gross Spread	0.09	0.09	0.03	0.07	0.10	0.00	0.20
Hot Issue	0.35	0.00	0.48	0.00	1.00	0.00	1.00

Table 4 presents the correlation matrix for the independent variables. None of the Pearson coefficient exceeds 0.389, which suggests that there may not be any serious multicollinearity problems. We find Pearson correlation statistical significance among the following relationships: the number of corporate executives with the healthcare background and firm size is positively associated, underwriter reputation and firm size is positively associated, firm size and gross spread is negatively associated and, underwriter reputation and gross spread is negatively associated.

**Table 4:** Pearson Correlation Matrix.

Pearson Correlation	GP	LNE	LUR	LNL	Hot Issue	Healthcare	Analytic	Biotechnology
GP	1.000							
LNE	−0.170**	1.000						
LUR	−0.389**	0.382*	1.000					
LNL	−0.052	−0.048	0.107*	1.000				
Hot Issue	−0.050	−0.072	0.162**	0.301**	1.000			
Healthcare	−0.092	0.283**	0.025	0.026	−0.110	1.000		
Genomic	0.031	0.126	0.015	0.129*	−0.045	−0.051	1.000	
Biotechnology	−0.012	0.103	0.041	−0.048	0.067	−0.074	−0.147**	1.000
Regulatory	−0.150**	0.124*	0.133*	−0.031	0.034	0.056	−0.144**	0.054

GP: Gross Spead; LNE: Log of Number of Employees; LUR: Log of Underwriter Reputation; LNL: Log of Number of Shares Locked Up;

** Correlation at 0.01(2-tailed statistical significance),

* Correlation at 0.05 (2-tailed statistical significance).

In table 5, model 3, none of the VIF factors exceeds 1.43. As such, multicollinearity is probably not a problem. Autocorrelation is probably not an issue because the Durbin Watson D statistics range from 1.74 to 1.75. The high adjusted r-square indicates that over 66% of the variation is explained in all OLS models. Thus, the models exhibit excellent fit by IPO research standards (see footnote 9).

**Table 5:** Multivariate Regression Tests.

Independent Variables	1	2	3	Variance Inflation Factor
(Constant)	(8.83)	(8.77)	(8.56)	
**Control Variables**				
Log of Number of Employees	0.362	0.402	0.388	1.38
	(8.76)***	(9.24)***	(9.08)***	
Log of Number of Shares Locked Up	0.122	0.146	0.150	1.15
	(3.06)***	(3.66)***	(3.83)***	
Log of Underwriter Reputation	0.266	0.254	0.250	1.43
	(5.93)***	(5.71)***	(5.76)***	
Gross Spread	−0.327	−0.329	−0.314	1.22
	(−7.89)***	(−8.04)***	(−7.81)***	
Hot Issue	0.325	0.308	0.304	1.16
	(8.05)***	(7.68)***	(7.74)***	
**Explanatory Variables**				
Healthcare		−0.083	−0.085	1.15
		(−2.08)**	(−2.17)**	
Genomic		−0.104	−0.085	1.11
		(−2.67)***	(−2.20)**	
Biotechnology		0.009	0.007	1.06
		(0.24)	(0.19)	
Regulatory			0.130	1.06
			(3.46)***	
Adjusted R-Square	0.66	0.67	0.68	
Durbin-Watson	1.81	1.80	1.80	

The variance inflation factors are for model 3. The t-statistics are in parentheses. The numbers without parenthesis are the standardized beta coefficients for each variable.

** Statistical significance at 5%,

*** Statistical significance at 1%.

We present the main results with the respective p-values in table 5. The control variables are all statistically significant and the signs are all in the expected directions. The firm size, hot issue dummy, underwriter reputation and the number of shares locked up are all positively associated with IPO proceeds while the gross spread is negatively associated with IPO proceeds. Further, these control variables remain statistically significant and the sign remain unchanged under sensitivity analysis.

We rejected the null hypothesis that the number of executives with healthcare industry background is not related to IPO net proceeds at 5% significance level. The results suggest that the number of executives with the healthcare industry background could be negatively associated with IPO proceeds. We also rejected the null hypothesis that the number of executives with the analytical/genomic tools industry background is not related to IPO net proceeds at 5% significance level. The results suggest that the number of executives with analytical/genomic tools industry background could be negatively associated with IPO proceeds.

However, we failed to reject the null hypothesis that the number of executives with the physician and/or pharmaceutical industry backgrounds is not related to IPO net proceeds. A possible reason is that our research design is not sophisticated enough to accommodate for “strategic games” played against the biotechnology companies. As mentioned in the industry analysis, healthcare establishments such as the HMOs face a dearth of growth options. However, they are in a powerful position due to the payment system, which require that the patients pay a healthcare fee upfront while the physicians claim reimbursement from the HMOs. Thus, the only way for the HMOs to maintain profits is to slow down the growth in healthcare spending. Prior research suggests that such actions tend to diminish the growth options of other life science companies in the upstream [76]. For example, increased HMO enrollment slows the diffusion of cardiac technologies, radiation therapies, diagnostic radiology, and extracoreal shock wave lithotripters [76]. This is a relevant consideration because our analysis of the IPO prospectuses indicates that some issuers list dependence on third party reimbursement as a risk factor. Further, such behaviors are expected whenever the intermediary (i.e. health insurer) is in a strong bargaining position [79]. That is, investors will be less willing to invest in biotechnology companies if insurers have strong bargaining power and can impose limits on the growth options of biotechnology companies.

We reject the null hypothesis that the number of executives with the regulatory industry background is not associated with IPO proceeds at 1% statistical significance. The results suggest that the number of executives with the regulatory industry background could be associated with IPO proceeds.

### Economic significance

In terms of economic significance, an increase of 0.99 regulatory elite (one standard deviation) translates to 4.4% change (beta coefficient) in the IPO net proceeds. An increase of 0.83 healthcare corporate elite and an increase of 0.79 genomic corporate elite both translate into a 2.9% decrease in the IPO net proceeds. Collectively, the explanatory variables account for 10.2% in the change of IPO net proceeds and should be considered as economically significant. All the control variables are also economically significant individually. A single standard deviation in the log of number of employees, log of number of shares locked up, log of underwriter reputation, gross spread and hot issue are each associated with 13%, 5%, 9%, 11% and 10% in the change of IPO net proceeds respectively.

### Sensitivity analysis

A potential research limitation is that there might be certain variables relevant for explaining both IPO net proceeds as well as our explanatory variables. Therefore, in table 6, we tested for omitted variable bias by controlling for independent variables that are potentially relevant.

**Table 6:** Sensitivity Analysis.

Independent Variables	4
(Constant)	(8.56)
**Control Variables**	
Log of Number of Employees	0.416
	(8.84)***
Log of Number of Shares Locked Up	0.130
	(3.30)***
Log of Underwriter Reputation	0.206
	(4.00)***
Gross Spread	−0.307
	(−7.54)***
Hot Issue	0.299
	(7.49)***
**Explanatory Variables**	
Healthcare	−0.093
	(−2.37)**
Genomic	−0.082
	(−1.94)**
Biotechnology	0.006
	(0.17)
Regulatory	0.138
	(3.51)***
**Sensitivity Variables**	
Firm Age	−0.050
	(−1.31)
JD/Ph.D/MD	−0.012
	(−0.27)
Number of VCs in the Board	0.035
	(0.81)
Venture Capital Invested	0.073
	(1.73)*
Spin Off	0.012
	(0.30)
NASDAQ	0.017
	(0.37)
Foreign Issuer	0.080
	(2.19)**

Firm age is computed as the difference between the founding date and IPO date. JD/Ph.D/MD refers to the number of executives in the firm with a Juris Doctorate, Medical doctorate or Ph.D. Venture capital invested refers to the amount of money invested in the firm by venture capitalists. VCs refers to venture capitalists. Spin off refers to the IPO of a subsidiary. Nasdaq refers to a IPO that is listed on the Nadaq stock exchange. Foreign issuer refers to a foreign company listing in the United States.

* Statistical significance at 10%,

** Statistical significance at 5%,

*** Statistical significance at 1%.

Given that the participation of venture capitalists and the educational qualification of the corporate elite might affect our results [80], [65], we control for the number of venture capitalists on the board, the amount of venture capital invested and the number of senior managers and directors with JD/PhD/MD. We control for firm age since Megginson and Weiss [69] suggested that younger firms tend to have more growth options. We control for whether the IPO firm is a foreign issuer because prior research suggest that foreign issuers are relatively high quality firms and enjoy a higher valuation or cross listing premium [40], [81]. Finally, we control for IPO characteristics such as whether the firm is a spin-off and/or listed in the Nasdaq stock exchange. We termed these additional variables as sensitivity variables.

Two sensitivity variables (venture capital invested and foreign issuer dummy) are weakly statistically significant and statistically significant respectively. The level of statistical significance and sign remains unchanged for both the control and explanatory variables after we include the additional sensitivity variables. In additional analysis, we use the gross proceeds and the market-to-book ratio as the alternative dependent variables and the results are qualitatively the same relative to the original functional specification.

We also incorporate a few key amendments into the regressions. First, we control for the average annual sales growth for each sector. Second, we control for EBITA (earnings before interest, tax and amortization) and R&D expenditures. Specifically, we control for the EBITA and the R&D expenditures for the calendar year prior to the firm going public. In addition, we seek to accommodate potential criticisms that the IPO proceeds need to be adjusted for inflation, and that the percentage rather than the number of executives with certain backgrounds (genomic, biotechnology, healthcare and regulatory) might be a more effective proxy.

The results are presented in table 7. To compute the average annual sales growth for each sector, we downloaded annual sales data from 1995 to 2001 for companies similar to each sector (genomic, biotechnology, healthcare and medical devices).

**Table 7:** additional sensitivity tests – 182 firms.

Independent Variables	5	6	7	8
(Constant)	3.175	3.117	10.843	10.783
	7.978)***	7.736)***	27.890)***	27.428)***
**Control Variables**				
Log of Number of Employees	0.305	0.322	0.305	0.323
	(6.055)***	(6.446)***	(6.022)***	(6.430)***
Log of Number of shares Locked up	0.104	0.112	0.104	0.113
	(2.384)**	(2.544)**	(2.370)**	(2.538)**
Log of Underwriter Reputation	0.042	0.046	0.042	0.046
	(0.703)	(0.761)	(0.697)	(0.754)
Gross Spread	−0.461	−0.460	−0.472	−0.471
	(−8.286)***	(−8.231)***	(−8.419)***	(−8.374)***
Hot Issue	0.294	0.284	0.269	0.258
	(6.501)***	(6.239)***	(5.929)***	(5.674)***
Sector Sales growth	0.117	0.116	0.117	0.116
	(2.629)***	(2.614)***	(2.608)***	(2.595)***
EBITA	0.073	0.070		
	(1.478)	(1.409)		
EBITA – Deflated			0.091	0.088
			(1.835)*	(1.769)*
R&D Expenditure	0.023	0.028		
	(0.464)	(0.560)		
R&D Expenditure - Deflated			0.015	0.020
			(0.306)	(0.393)
**Explanatory Variables**				
Healthcare – Number	−0.092		−0.094	
	(−2.288)**		(−2.319)**	
Healthcare - Percentage		−0.095		−0.097
		(−2.330)**		(−2.370)**
Genomic – Number	−0.108		−0.107	
	(−2.307)**		(−2.274)**	
Genomic – Percentage		−0.121		−0.120
		(−2.524)**		(−2.490)**
Biotechnology – Number	0.051		0.055	
	(1.236)		(1.318)	
Biotechnology - Percentage		0.028		0.033
		(0.674)		(0.793)
Regulatory – Number	0.128		0.129	
	(2.913)***		(2.912)***	
Regulatory – Percentage		0.117		0.120
		(2.766)***		(2.812)***
**Sensitivity Variables**				
Firm Age	−0.024	−0.026	−0.027	−0.028
	(−0.569	(−0.595	(−0.622)	(−0.647)
JD/Ph.D/MD	−0.064	−0.041	−0.058	−0.035
	(−1.261	(−0.832	(−1.122)	(−0.690)
VC Factor	0.120	0.114	0.122	0.117
	(2.373)**	(2.221)**	(2.400)**	(2.264)**
Spin Off	0.054	0.055	0.058	0.059
	(1.069)	(1.079)	(1.136)	(1.156)
NASDAQ	0.008	0.002	0.008	0.002
	(0.158)	(0.046)	(0.151)	(0.041)
Foreign Issuer	0.086	0.080	0.083	0.076
	(2.170)**	(1.984)**	(2.066)**	(1.883)*
Adjusted R-Square	0.732	0.729	0.728	0.726
Durbin-Watson D Statistics	1.722	1.711	1.773	1.764

Ordinary least squares regressions of the log of IPO net proceeds on explanatory variables denoting proxies for various investment strategies, a set of control variables and a set of sensitivity variables. The dependent variable in Model 5 and 6 is the log of IPO net proceeds whereas the dependent variable in Model 7 and 8 is the log of IPO net proceeds deflated to 1967 dollars. The control variables are gross spread, log of the number of employees, log of underwriter reputation, log of the number of shares locked up, hot issue and sector sales growth. Gross spread is measured as the difference betIen IPO gross proceeds and IPO net proceeds over the IPO gross proceeds. The number of employees is downloaded from SDC. The measure of underwriter reputation is based on a ranking table provided by Loughran and Ritter [5]. The “number of shares locked up” is downloaded from SDC. Hot issue is coded as 1 if the IPO occurs in 1999 or 2000 and 0 otherwise. Sector sales growth is the change in average annual sales for the genomic, biotechnology, medical devices and healthcare sector. EBITA is the earnings before interest, tax and amortization expenses in the year that the issuer go public. R&D expenditure is research and development expenditure and is downloaded from Compustat. EBITA-deflated refers to EBITA deflated to 1967 dollars. R&D expenditure-deflated refers to R&D expenditure deflated to 1967 dollars. The explanatory variables in model 5 and 6 are number of corporate elites with healthcare industry background while the explanatory variables in model 7 and 8 are percentage of corporate elites with healthcare industry background. Healthcare industry background refers to experience in selected areas such as home health care services, hospitals, outpatient care centers, nursing and residential care facilities and, HMOs and health/medical insurers. We report standardized beta coefficients for all variables except for constant. I report beta coefficient for the constant. T-statistics are in parentheses. Statistical significance at 10%, 5% and 1% are denoted respectively as *, ** and ***.

We first computed a simple average of sales for each sector and each year as
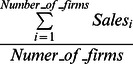
(2)Then, we calculated the sector sales growth for each year as
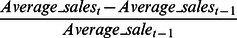
(3)The data on EBITA and R&D expenditure are downloaded from Compustat. Because data on EBITA (calendar year prior to the firm going public) is missing for 40 companies, we reduced the sample further to 200 companies. Within the sample of 200 companies, we found that data on R&D expenditure (calendar year prior to the firm going public) is missing for 18 companies. Therefore, the sample is further reduced to 182 companies.

In model 5 and 6, we continued to use the original dependent variable where the IPO proceeds are not adjusted for inflation. In model 7 and 8, we deflated the IPO proceeds along with the EBITA and R&D expenditure. The CPI published by the Bureau of labor statistics uses 1967 as the base year. Therefore, we deflated the monetary amounts to 1967 dollars.

In model 5 and 7, our four explanatory variables were the number of executives with healthcare, genomic, biotechnology and regulatory industry backgrounds. In model 6 and 8, our four explanatory variables are the percentage of executives with healthcare, genomic, biotechnology and regulatory industry backgrounds. The results remain qualitatively unchanged (see footnote 10).

## Conclusions

Our specific contribution lies in the application of the upper echelon theory to the IPO phenomenon. By presenting an “industry background” explanation to IPO net proceeds, we highlight the fact that the relationship between IPO proceeds and underpricing cannot be simply assumed. In developing our hypotheses, we argued that investors make inferences about the investment strategies and growth options that the firm is likely to exercise based on the corporate executives' industry backgrounds. While empirical evidence on the explanatory power of growth and/or real options started to emerge recently [82], recent research did not apply the upper echelon theory to understand how real options reasoning could be applied to IPOs. By contrast, we use upper echelon theory to explain how investors might use real options reasoning to relate senior executives' industry backgrounds to the investment strategies and growth options of IPO firms. Specifically, by applying the upper echelon theory to the IPO phenomenon, our findings contribute to the subfield of research that arises from the intersection between the literatures of IPO studies and upper echelon theory. The findings are important because upper echelon theorists have stated that considerably more research on the relation between executives' demographic variables and organizational outcomes are needed [83]. Likewise, resource dependence theorists have lamented that the resource dependence theory is much cited but not frequently tested [84]. However, our approach goes one step further. In addition to framing the ability to extract resources from the government as a real or growth option (i.e. a firm can leverage on its ability to interact with the government to grow), we also argued that top managers with legal and regulatory backgrounds have the ability to extract resources from the government. This approach integrates the resource dependence theory into the upper echelon theory.

In summary, we identified four unique variables for empirical testing. The choice of proxy variables and their relation to the investment strategies is based on the upper echelon theory and the relevant real options literature. Empirically, the relationship between the proxy variables and IPO proceeds are statistically significant except for the biotechnology variable.
